# Supplementary material for: Experimentally induced metamorphosis in highly regenerative axolotl (ambystoma mexicanum) under constant diet restructures microbiota
Source: Sci Rep. 2018 Jul 20;8:10974. doi: 10.1038/s41598-018-29373-y (PMC6054665; doi:10.1038/s41598-018-29373-y)

Supplemental Figures (S1 to S11)

Experimentally Induced Metamorphosis in Highly Regenerative Axolotl (*Ambystoma mexicanum*) Under Constant Diet Restructures Microbiota

Turan Demircan[1,6*], Guvanch Ovezmyradov[2,6] , Berna Yıldırım[6], İlknur Keskin[3,6], Ayşe Elif İlhan[6], Ece Cana Fesçioğlu[6], Gürkan Öztürk[4,6], Süleyman Yıldırım [5,6*]

Supplementary Figure S1. Time course of metamorphosis and limb regeneration. Time course (Day0 -Day72) after 6 weeks of T4 administration and additional 1 month beyond hormone treatment. The figure shows anatomical changes to adapt terrestrial life conditions. Metamorphosis-associated characteristics such as weight loss and disappearance of fin and gills were noticed gradually within this time period.

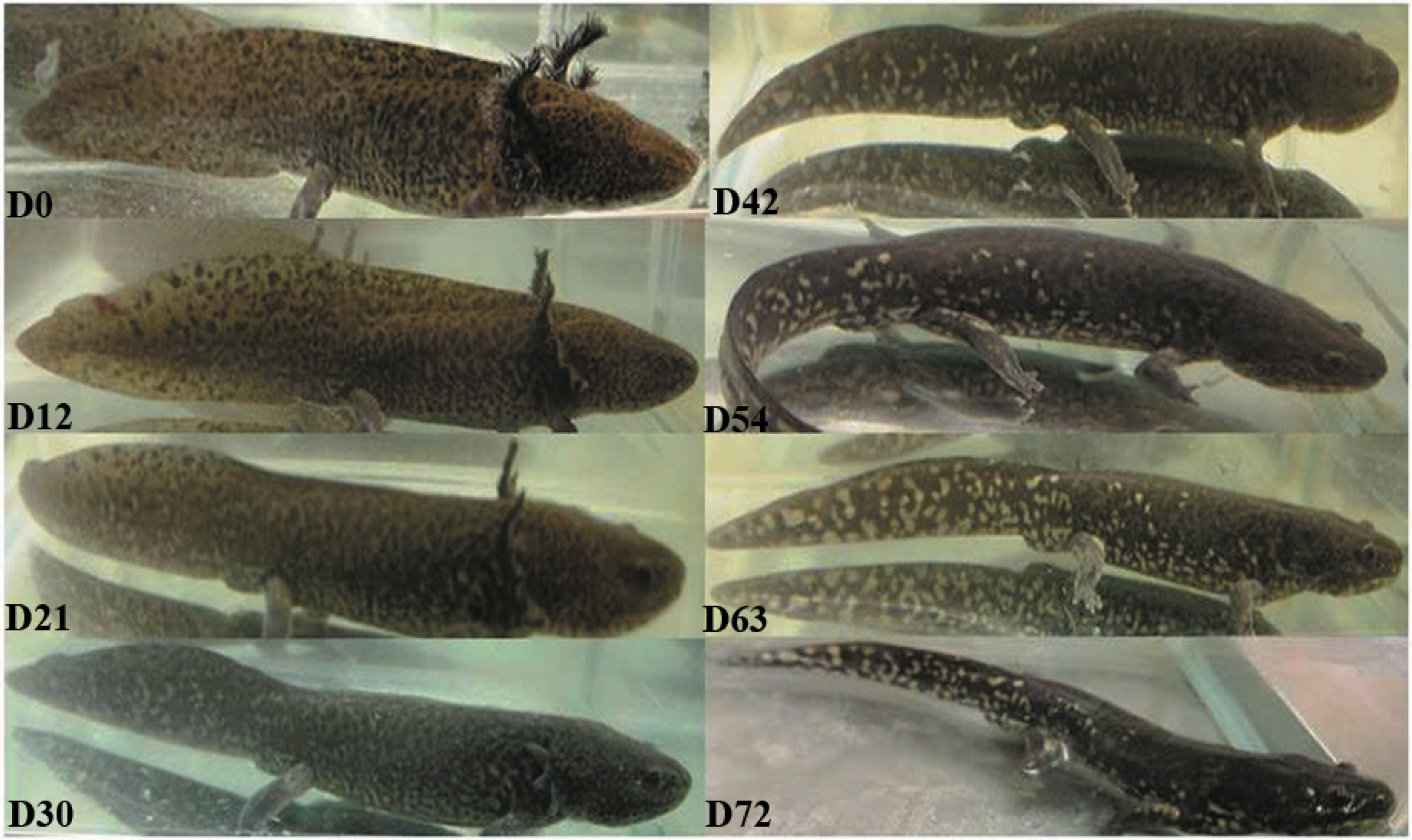

Supplementary Figure S2. Phylogenetic relationship of *denovo* OTUs identified in this study with related sequences in NCBI (nr) database. MOLE-BLAST was used to search and obtain the phylogenetic tree based on minimum evolution algorithm. Only 200 OTUs are shown for simplicity. Results from “Sequences from type material” run are shown in the second page highlighted by green color.

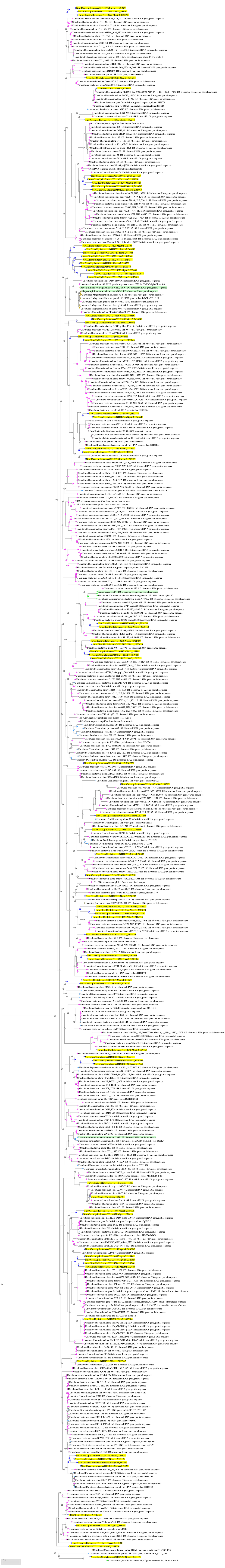

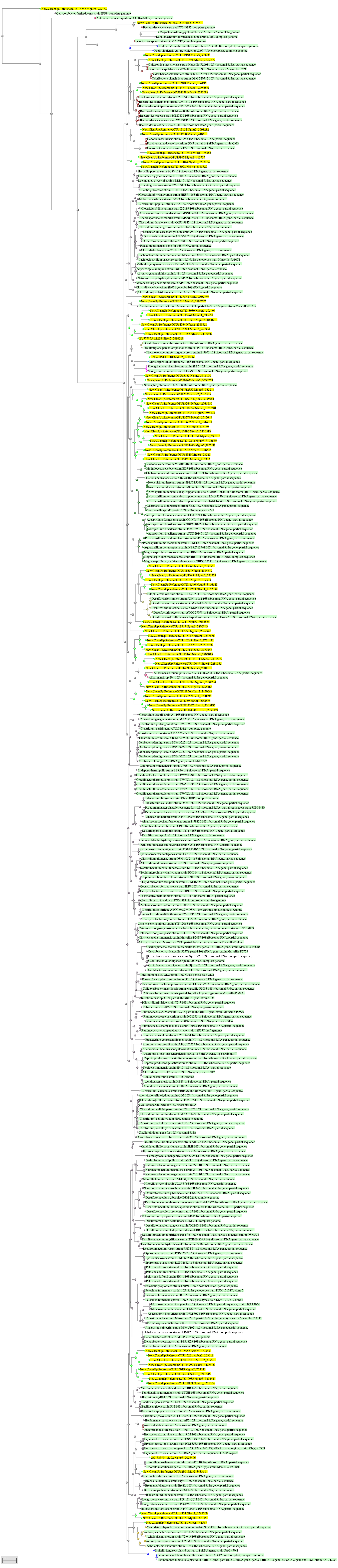

Supplementary Figure S3. Principle Coordinates Analysis (PCO) plot based on Jaccard distance matrix showing clustering of studied bacterial communities

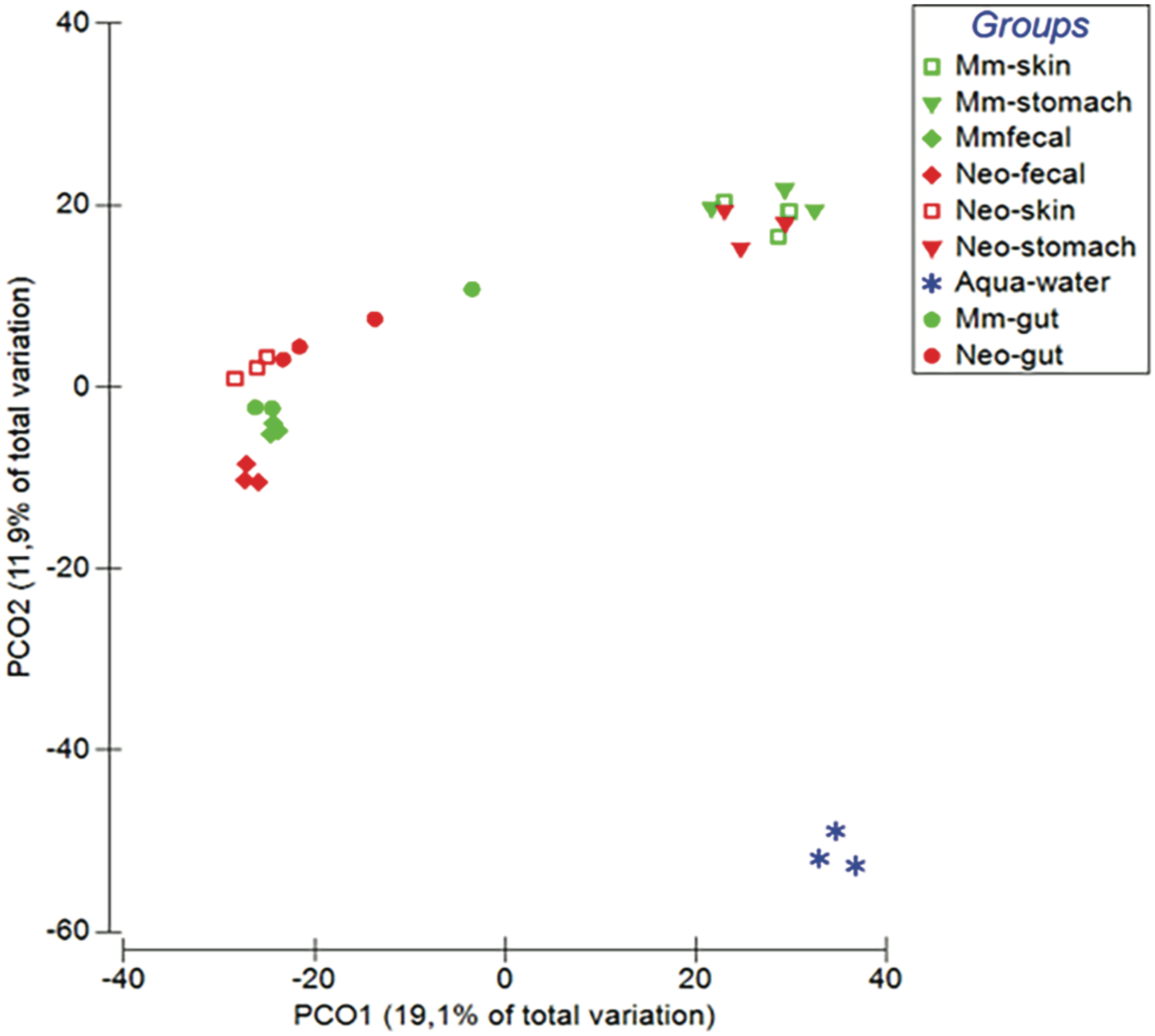

Supplementary Figure S4. Phylum-level correlation heatmap of OTU abundances based on Pearson's correlation coefficient

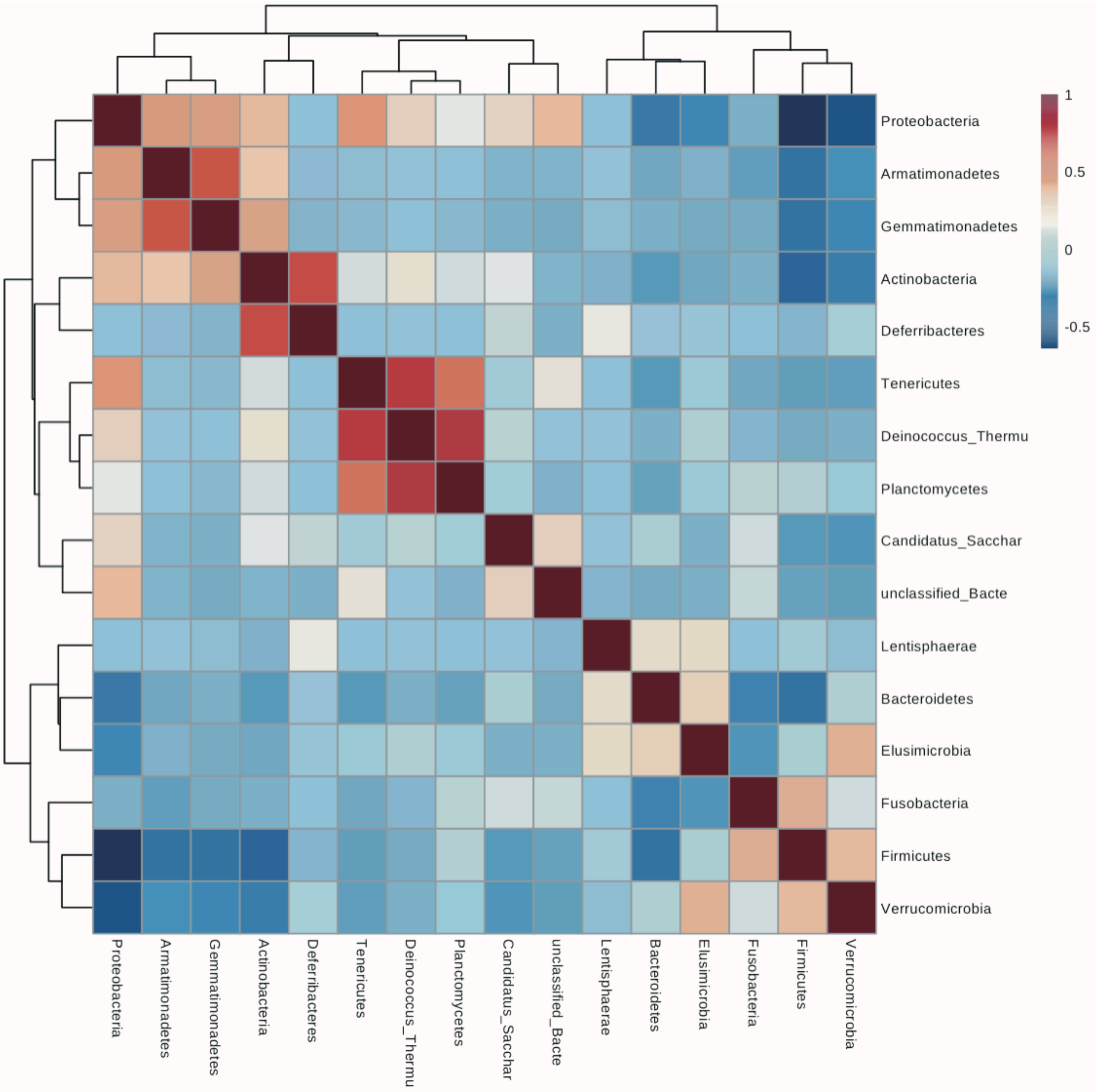

Supplementary Figure S5a and S5b. Venn diagrams showing the number of unique and shared OTUs between skin, stomach, gut, and fecal samples of neotenic (a), and metamorphic Axolotl (b).

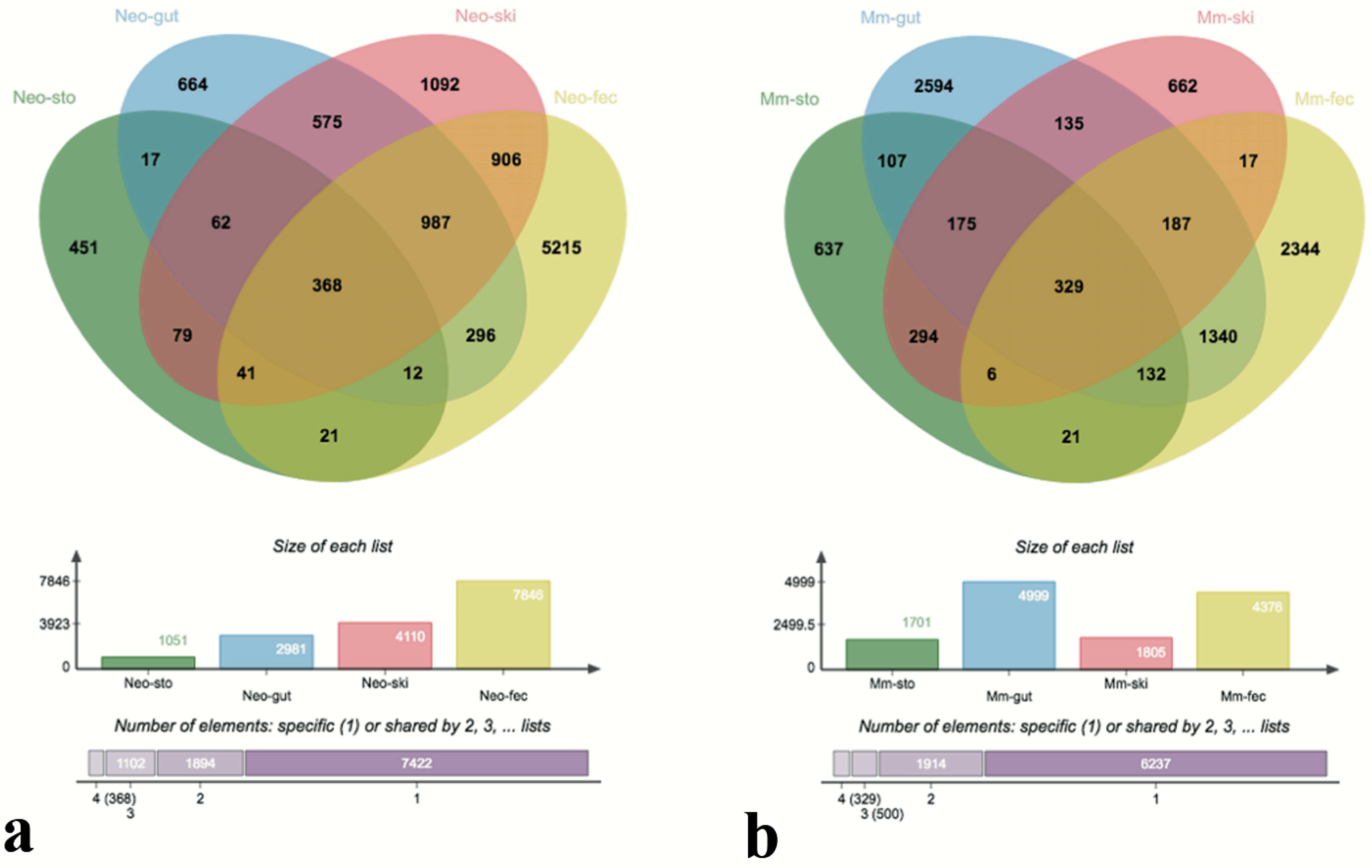

Supplementary Figure S6. Heatmap showing core OTUs (Core90) at varying detection threshold and percent abundance. The color scale bar shows prevalence of OTUs.

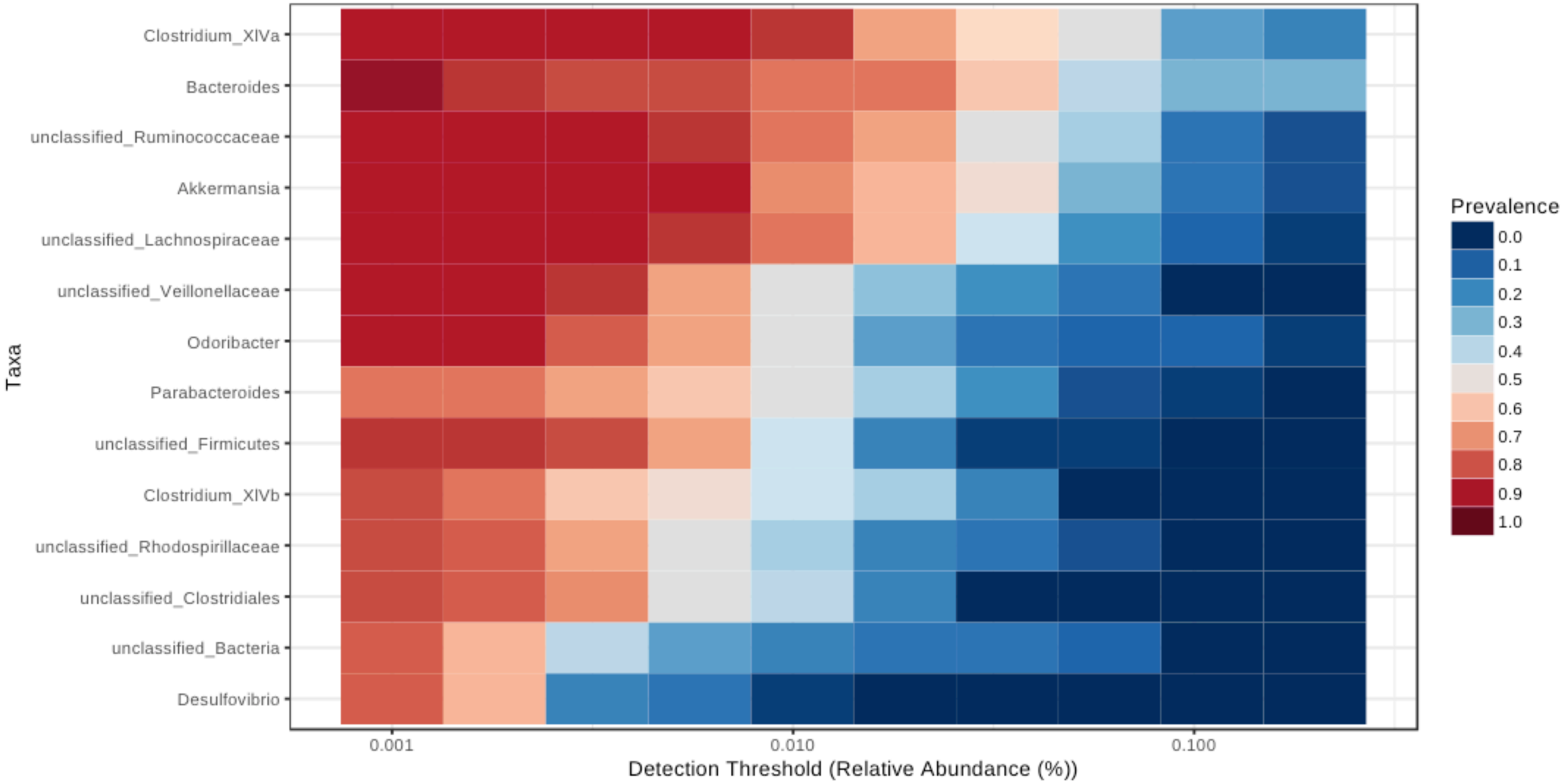

Supplementary Figure S7a. Comparison of Axolotl gut microbiota with Humans gut microbiota based on HMP data. Principle Coordinate Analysis (PCoA) uses unweighted UniFrac distance.

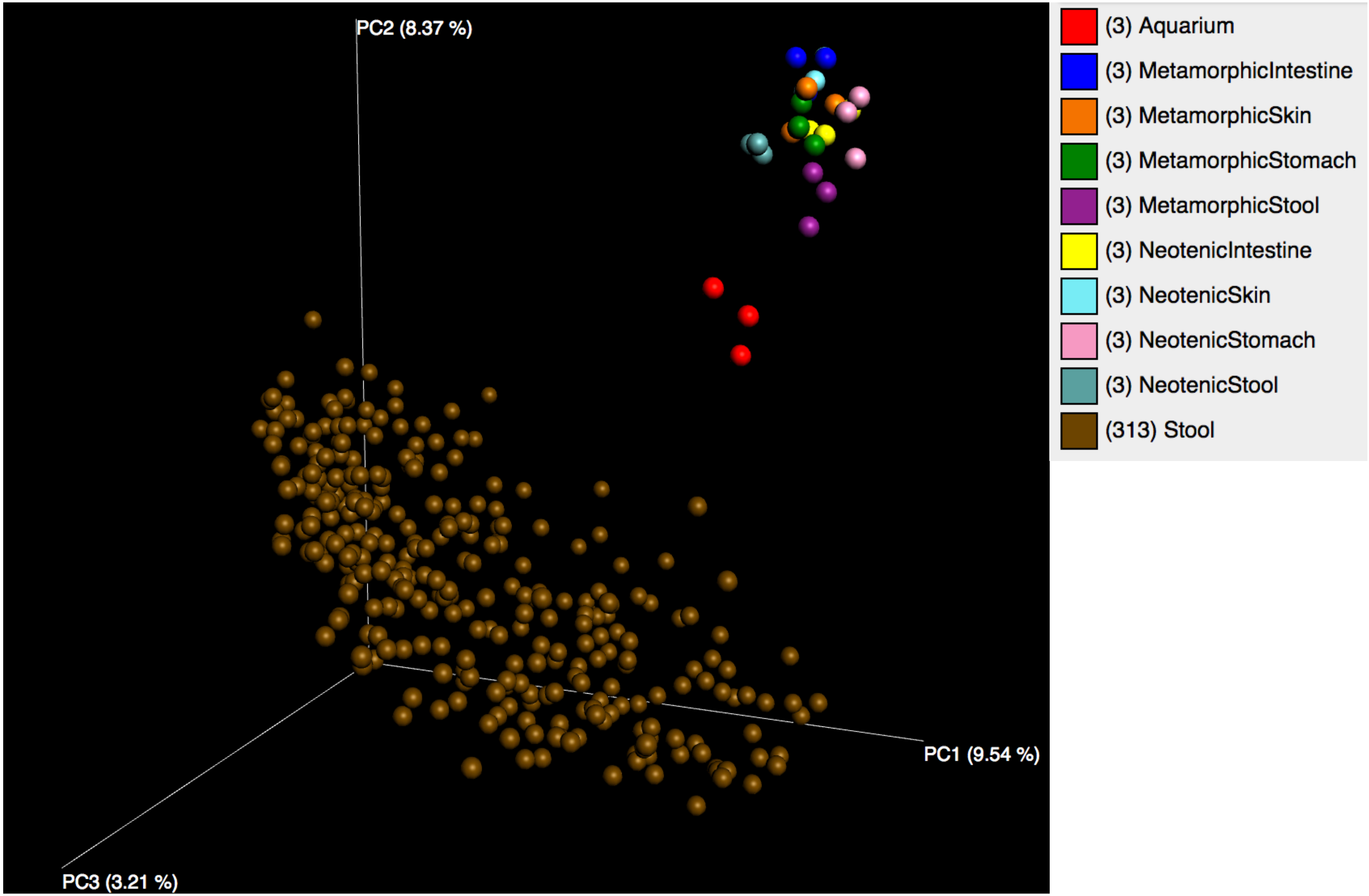

Supplementary Figure S7b. Comparison of Axolotl gut microbiota with Humans gut microbiota based on HMP data. Principle Coordinate Analysis (PCoA) uses weighted UniFrac distance.

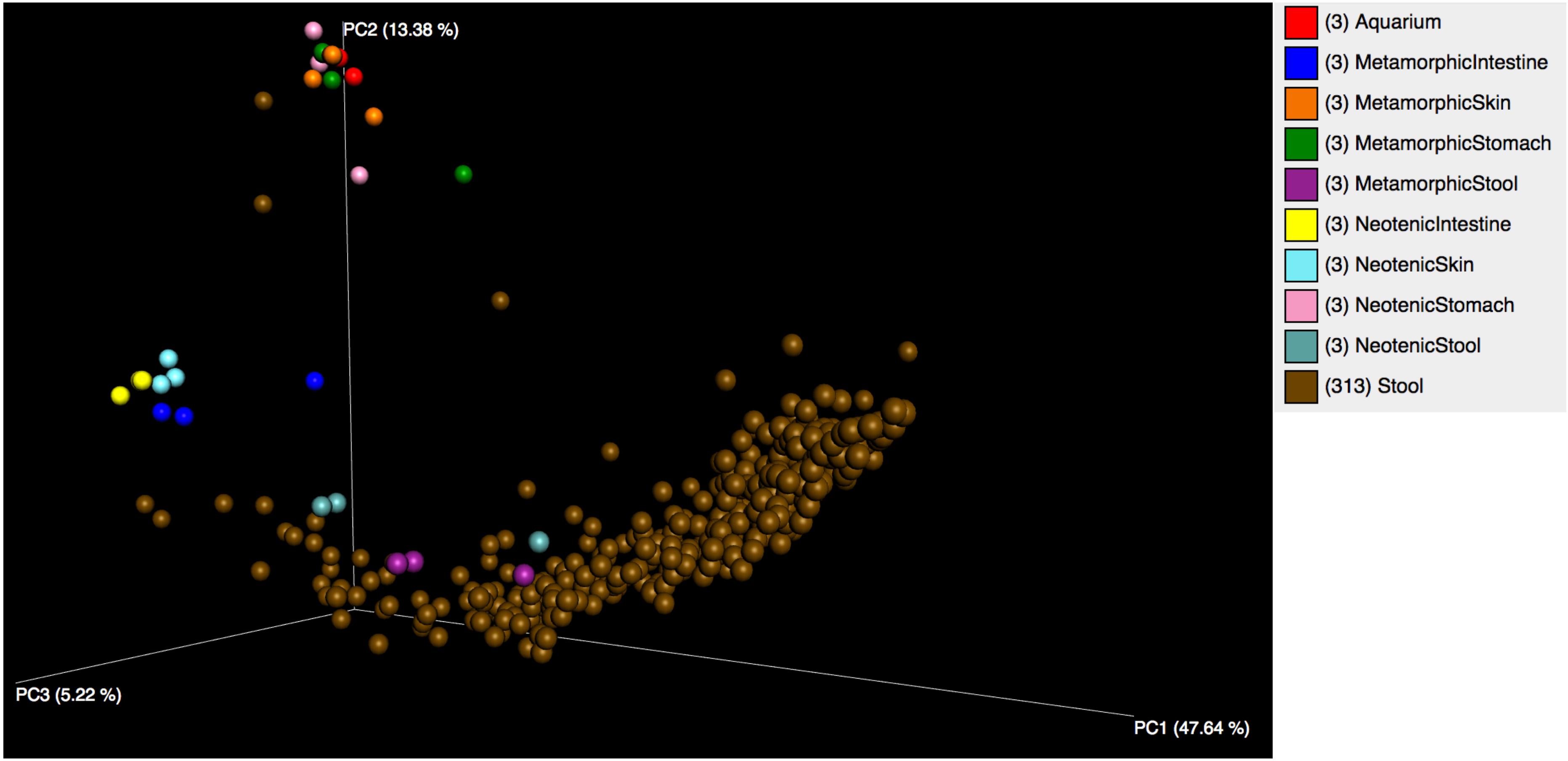

Supplementary Figure S7c. Comparison of Axolotl skin microbiota with Humans skin microbiota based on HMP data. Principle Coordinate Analysis (PCoA) was constructed using unweighted UniFrac distance.

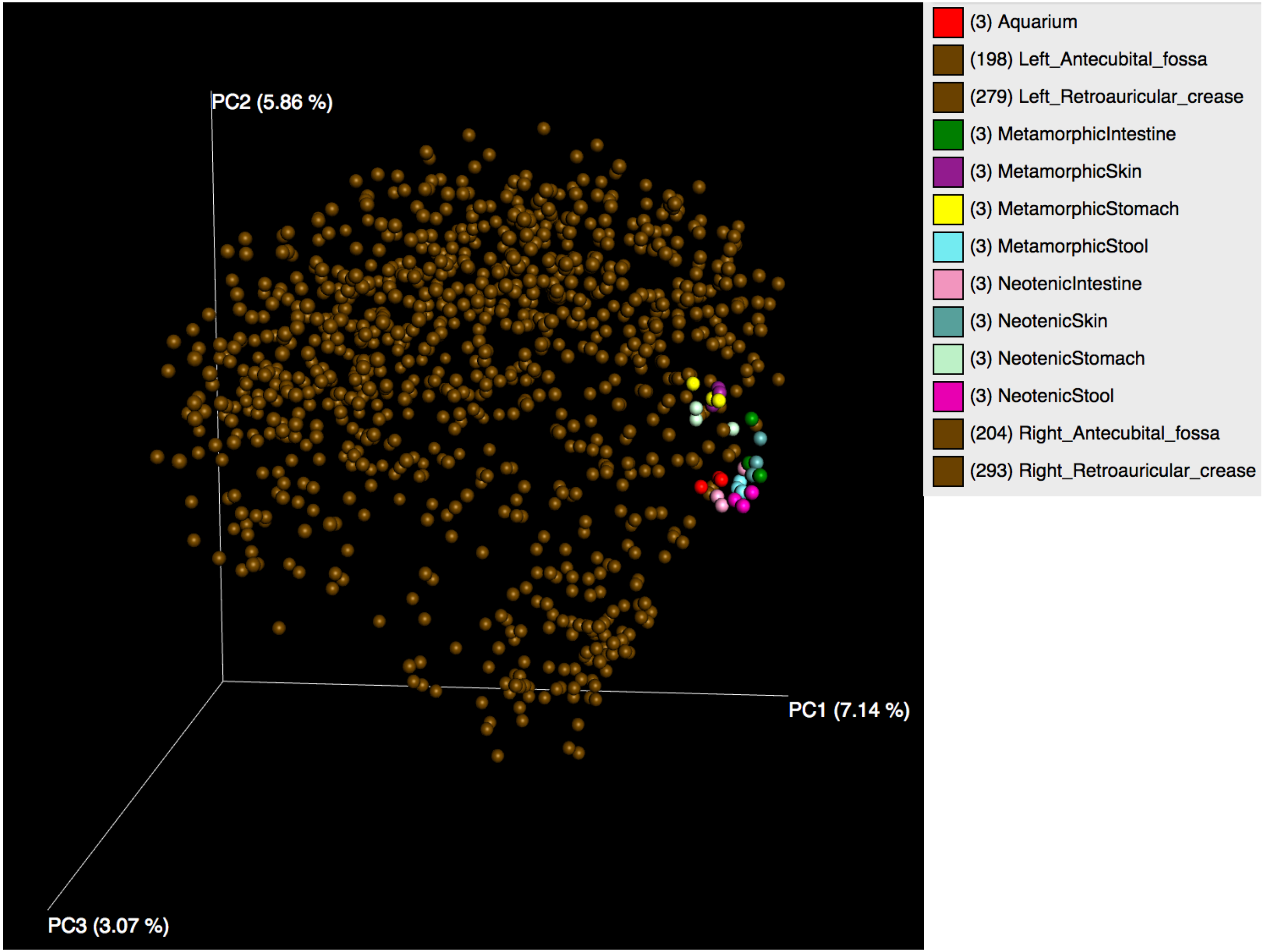

Supplementary Figure S7d. Comparison of Axolotl skin microbiota with Humans skin microbiota based on HMP data. Principle Coordinate Analysis (PCoA) was constructed using weighted UniFrac distance.

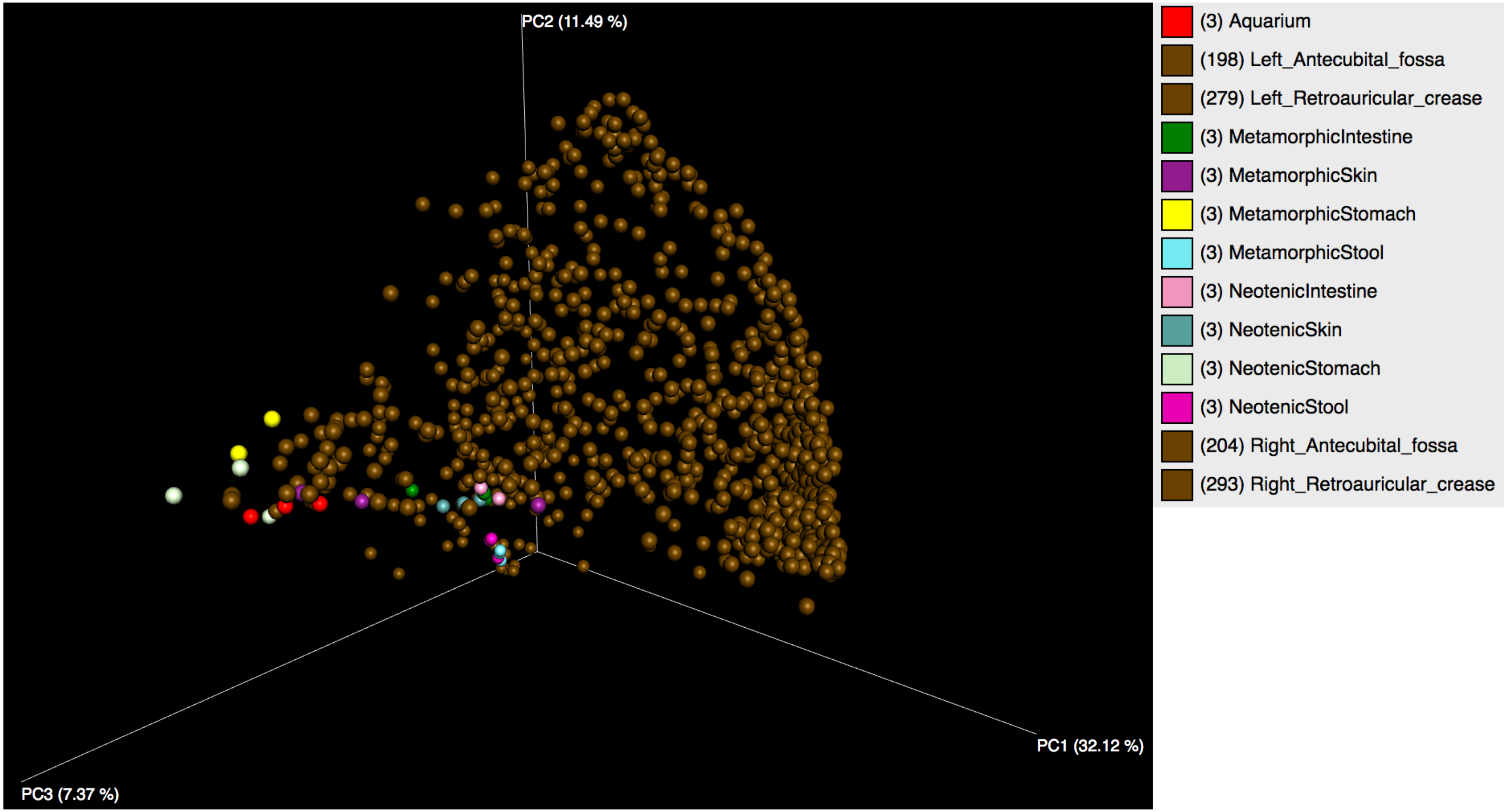

Supplementary Figure S8. Canonical Analysis of Principal Coordinates (CAP) analyses of predicted functional abundances of gut bacterial communities from Axolotl samples based on Bray-Curtis distance matrices.

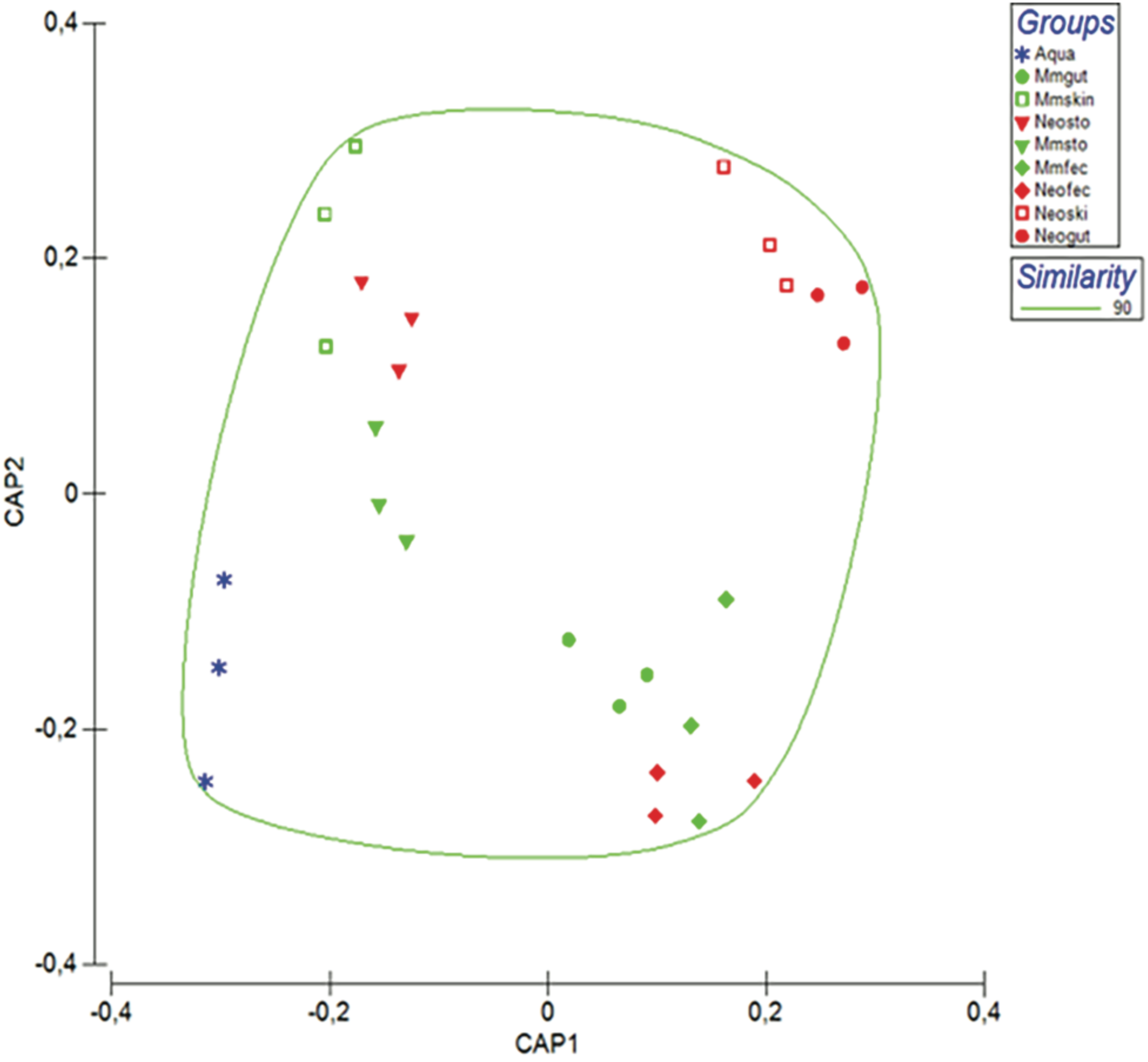

Supplementary Figure S9. Histological comparison of neotenic and metamorphic gut tissue. Neotenic (A–C) and metamorphic Axolotl's (A'–C') intestine sections demonstrate structural similarities rather than differences. Mucus producing goblet cells were more abundant in metamorphic samples, which was also accompanied by a thicker mucosa layer. *: goblet cells, Scale bar = 100 m

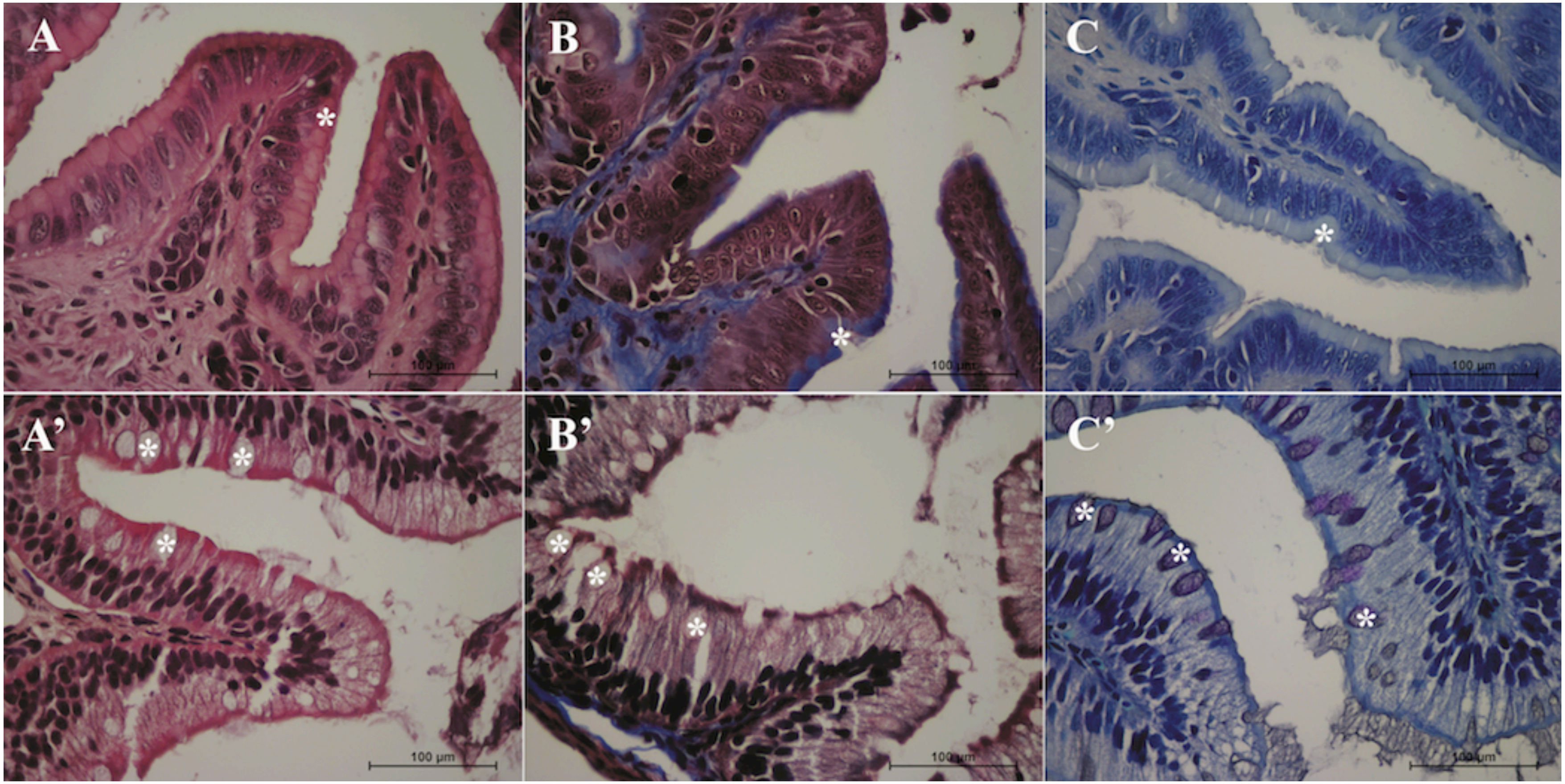

Supplementary Figure S10. Time course (Day0 - Day64) after limb amputation demonstrating differences between regenerative capacity of neotenic (upper panel) and metamorphic (lower panel) axolotl. Reduction in limb regenerative capacity was observed for metamorphic animals. (n=15 for each group) .

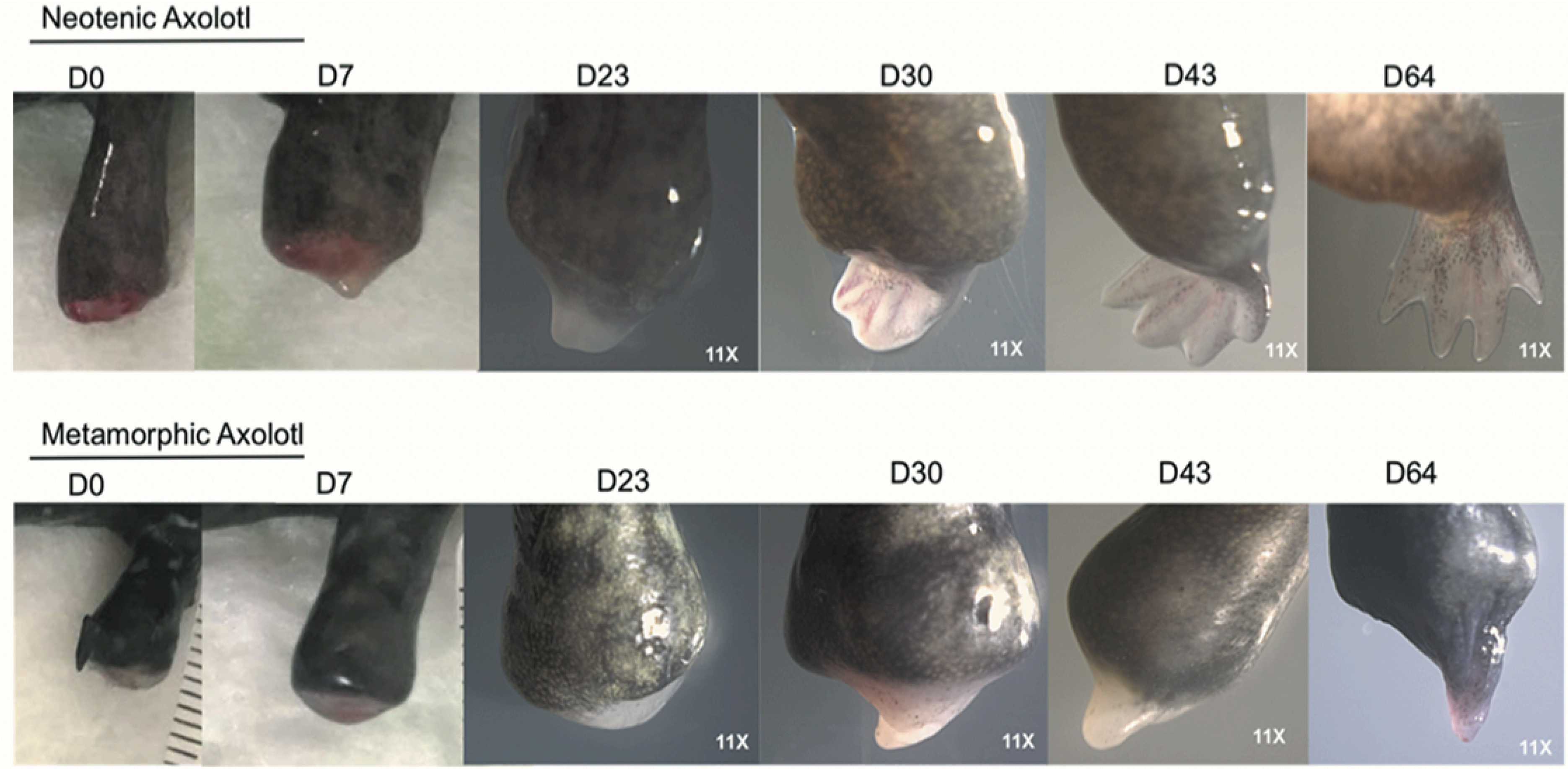

Supplementary Figure S11 (a-c). Metamorphosis constrains Axolotl regeneration capacity and fidelity (a-c). Representative images of the limbs at the end of experiment, at day 150, for successful (a-a') and unsuccessful limb regeneration (b-b' and c-c').

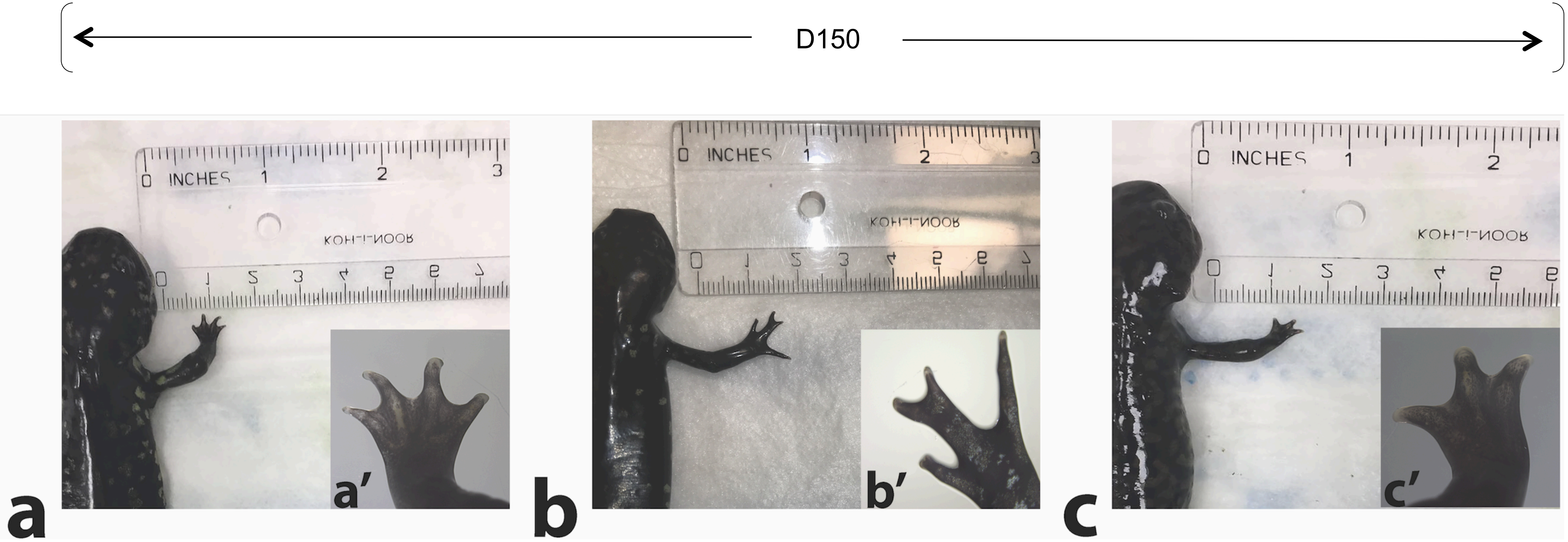

Supplement: Supplementary file 1 — Supplementary Information [file 41598_2018_29373_MOESM1_ESM.pdf]
